# Social class, marginality and self-assessed health: a cross-sectional analysis of the health gradient in Mexico

**DOI:** 10.1186/1475-9276-8-3

**Published:** 2009-02-23

**Authors:** Adolfo Martinez Valle

**Affiliations:** 1 Formerly Secretaría de Salud, Mexico City, Mexico

## Abstract

**Background:**

Examining the association between social inequality and health is not new. However, there is little empirical evidence of this association in the Latin American literature, much less from the Mexican scholars. Its research, including the one conducted in Mexico, has mostly followed a theoretical approach and has not been able to provide strong empirical evidence of their important theoretical and conceptual contributions, mainly because reliable, complete and valid data are unavailable.

**Methods:**

To empirically examine the gradient effect of social class on self-rated health in Mexico, a secondary cross-sectional mixed-level analysis was designed. Using individual level data from the Second National Health Survey (ENSA II), social class categories were specified following a stratification approach according to the occupation and education indicators available from ENSA II. Two types of categories were made, one for t urban and one for the rural labor force. Two indicators of perceived health status were used as health outcomes: self-assessed health and reported morbidity. Furthermore, the marginality index, an indicator of relative deprivation was used to examine its contextual effect at the state and regional level. The analysis was conducted using logistic multivariate models.

**Results:**

The cross-sectional analysis showed a gradient effect of social class for good assessed-health. Relative to the low urban class, the odds ratio (OR) for a good perception of health for individuals belonging to the high urban class was 2.9 (95% confidence interval: 2.1–3.9). The OR for the middle high class was 2.8 (95% confidence interval: 2.4–3.4), while the OR for the middle low class was 1.8 (95% confidence interval: 1.6–2.1). However, for the rural labour force an OR of 1.5 was only significant between the high class who considered their health as good relative to the low class (95% confidence interval: 1.02–2.2). At the aggregate level, the results also showed individuals living in deprived regions were less likely to report their health as good than individuals living in relatively less deprived ones, OR = 0.6 (95% confidence interval: 0.4–0.7).

**Conclusion:**

Overall, the findings of this study provided empirical evidence that social inequality negatively influences health through a differential exposure and an unequal distribution of resources across the class spectrum: the lower the social class, the poorer the perception of health. The results also showed that living in more deprived regions had a further negative effect on health. From a policy perspective, the gradient effects of social class suggest that non-targeted policies should be designed to address both material conditions at the individual level as well as deprived living conditions at higher levels of aggregation to improve health across the social spectrum.

## Background

Examining associations between social inequality and health is not new [1-12]. Few empirical studies, however, have appeared in the Latin American literature; very few from the Mexican scholars. With a few exceptions, [13-17] Latin American research has followed a theoretical approach, providing little empirical evidence to support conceptual contributions [18-20].

Reliable, valid, and complete data are rare in Latin America, explaining the absence of empirical studies. National health surveys, conducted irregularly because they are expensive, constitute the best sources of information. These surveys inadequately address the social determinants of health, usually collecting only income and education data. Working with data collected in the latest national representative household health survey (ENSA) in 1994, this study examines the influence of social inequality on health in Mexico employing logistic multivariate statistical models.

Social inequalities, defined as the differences among social groups and the lack of social cohesion this differences create, may influence both individual and population health through two mechanisms. The first mechanism is derived from larger social, political and economic processes that shape the distribution of education, occupation and income across the population. This processes sort individuals into social class positions according to their control over different types of resources [21-24].

Social class positions are associated with health damaging "exposures"-diet, environmental hazards, and dangerous working conditions; access to social resources such as medical care, sewage systems, and drinking water; and individual resources such as income and education that reflect differential opportunities [21-24]. Surely individuals in lower social classes are more likely to experience negative exposures and to be more deprived of "health protective resources", but exposure and deprivation are not confined to the lowest social class. Their impact on health shows a gradient pattern-the lower the social class position, the higher the adverse health effects. Thus, social inequality can be expected to influence health negatively through a differential exposure and unequal distribution of resources across the class spectrum.

The second mechanism through which social inequality negatively influences health is related to how power is distributed in a society and how this distribution in turn shapes public policies. This study analysed the effects on health of living in relatively deprived areas as shaped by public policies. Living in relatively more deprived areas is the result of political processes that influence how public resources are distributed. Greater social inequality often accompanies a higher concentration of political power in the hands of the higher social classes who, in turn, demand reduced taxes and do not, in general, benefit from increasing public services [25-29]. The lower classes, in turn, are in a weaker political position and therefore face more constraints when articulating their demands and defending their interests [30-35].

In Mexico, health differentials associated with social inequalities have widened in the past two decades. The risk of dying for children under 5 years of age living in rural areas increased from 20 percent in 1992 to 55 percent in the year 2000 relative to children living in urban areas. Poverty remained relatively stable between 53 percent in 1984 and 55 percent in 1994, the year ENSA II was conducted. However, it grew considerably to 69 percent of the total population in 1996 due to an economic crisis that struck Mexico in 1994 [36]. According to the marginality index, an area-based indicator of relative deprivation, marginality has been reduced in 17 of the 32 Mexican states between 1990 and 1995, while the remaining 15 states have shown higher deprivation. However, from 1995 to 2005 marginality increased in 16 states, 11 remained practically unchanged and 10 diminished [37]. The relatively more deprived populations have worse health outcomes derived from their relatively poorer living conditions. For example, the risk of neonatal mortality in 2005 was 2.3 higher in the more deprived states of Chiapas and Oaxaca than Nuevo León, the least deprived one.

Political participation by the lower income classes, however, has created pressures for governments to respond with supportive public policies [34,35]. In the early 90s, government implemented a social safety-net program, the National Solidarity Program, now *Oportunidades*, combining conditional cash transfers with health, nutrition, and education assistance. The program has tried to improve living conditions for targeted vulnerable populations in the short term, while fostering capacity development in the medium term, by creating incentives to increase school attendance and regular use of preventive health services. Despite its growing coverage, and given the magnitude of poverty, the regional distribution of the funds, as well as the political criteria to allocate resources that favoured groups with greater potential for collective action, studies suggest that this targeted design of social policy was not very effective reducing poverty during the 1990s when the survey was conducted [36].

In sum, this paper aims to empirically examine the gradient effect of social class on self-rated health in Mexico, as well as analyse the effects on health of living in differential deprived areas. It also seeks to identify policy options to address this form of social inequality to improve the Mexican population health.

## Methods

To examine the association between social inequality and health, I conducted a cross-sectional, secondary analysis of the Second Mexican National Health Survey (ENSA II) using logistic multivariate statistical models and STATA 10. The overall ENSA II response rate was approximately 97 percent [38,39]. ENSA II, a national survey representative of the Mexican population at both the national and the regional level, contains information on health status and health services utilization of individuals, as well as demographic and socio-economic characteristics of households and individuals. Occupational data are needed to determine social class, and although nearly 15 years old, ENSA II from 1994, is the most recent national survey containing occupational data so that this study might measure social inequalities by stratifying according to social class. Mexico conducted two more recent surveys, in 2000 and 2006. These later ones emphasized the health component-taking blood samples to measure cholesterol and glucose levels, for example-and omitting important socio-economic information such as occupation.

To study the social class gradient in *self-assessed health*, the sample is restricted to the working population between 12–75 years of age. This age range reflects the official definition of the *economically active population *[40]. My sample was divided in two groups: the urban workforce-those who worked in urban settings-and the rural workforce-those engaged in agriculture-related activities, including the forestry and livestock industries. The dichotomy reflected my exploratory data analysis and previous studies [41,42] that suggested different socio-economic living conditions. The rural poor face much worse housing conditions than the urban poor. Approximately 30 percent of the rural households lack sanitary facilities, whereas only 3 percent of the urban households.

Self-rated health has been widely used to research health inequalities in developed western societies, but few such studies are available in developing countries. I used two indicators of perceived health status as health outcomes in this study: self-assessed health and self-reported morbidity. The social class gradient literature has shown that these indicators are sensitive to the effects of social inequality [11,43-45]. Self-rated health was originally rated on a 5-point scale: very poor, poor, average, good, and very good. For the purposes of this study, I transformed it to a dichotomous measure: equal to 1 if the response was average, poor, or very poor. Reported morbidity, reflected whether the respondent reported any health problems in the past two weeks: an illness, complaint or accident. In my study, reported morbidity also became a dichotomous variable equal to 1 if the respondent reported any health problem and otherwise 0.

Social class specification was done to better measure the health gradient. ENSA II does not include straightforward measures of social class membership. In many studies, several measures of socio-economic status have been used simultaneously, as a single measure has not adequately captured the health effects of social inequality [3,9]. In this study, social class has been constructed by combining two measures of social stratification present in ENSA II: education and occupation. Education was measured by years of schooling, while occupation was a multi-category discrete variable (See Tables 1 and 2). Based on possible combinations of these categories, four categories were constructed for the urban sector and three for the rural sector. High urban class comprises individuals whose occupation is a boss, an independent professional, or an employee of the manufacturing or services sector with at least a college degree. Low social class for both urban and agriculture sectors became the reference categories.

**Table 1:** Urban social class categories

**Variable**	**Occupation**	**Education**	**N (%)**
High	Employer Independent professional Employed	15 or more years 15 or more years	631 (4)
Middle high	Employed Non-salaried worker Salaried worker	9–14 years 9–14 years 9–14 years	2,500 (16)
Middle low	Employed Non-salaried worker Salaried worker	7–8 years 7–8 years 7–8 years	3,667 (24)
Low	Non-salaried worker Salaried worker	6 or less years 6 or less years	8,331 (56)

**Table 2:** Agricultural social class categories

**Variable**	**Occupation**	**Education**	**Income**	**N (%)**
High	Land owner Self-employed	10 years or more	6 goods	287 (6)
Middle	Land owner Salaried worker Self-employed	6–9 years	3–5 goods	952 (19)
Low-income	Land tenants Salaried worker Self-employed Non-remunerated workers	5 or less years	0–2 goods	3,613 (75)

Other variables associated with morbidity were included to control for its effects. Age was transformed to correct for its skewed distribution. Except for age, which is a continuous variable, all others were dichotomous variables. Age showed a highly skewed distribution, thus logarithmic transformation was used to specify the correct functional form. Gender was assigned 1 for a man. To compensate for the high non-response rate about income, I devised a proxy for income, based on the number of durable goods each individual possessed: automobile, television, video cassette player, refrigerator, gas stove, and water heater. Four variables were assigned: one, if an individual had all six items; two, if it had 4–5; three, if it had between 2–3; and four, the reference category, if he or she had 0–1. The reliability of this variable has been described elsewhere [46].

The National Population Council (CONAPO) supplied the *marginality index*, an aggregated measure [40] developed to measure the degree of marginality in each Mexican state and county. The index reflects deprivation based on housing, income, and schooling information collected from the 1990 Mexican Census and the 1995 Population and Housing Count [47,48]. The housing component refers to the percentage of people living in households in a town of less than 2,500 inhabitants, lacking running water, electricity, solid floor materials and sewage facilities as well as overcrowded living conditions. Education measures the percentage of illiterate people older than 15 years and percentage of people who did not finished the six years of basic education. The income component refers to the percentage of the economic active labor force earning less than twice the minimum wage, which is equivalent to 6 US dollars per day. CONAPO performed principal component statistical analysis to assess its validity. This index is a normalized Z-score ranging between -3 and 3 standard deviations that correspond to very low and very high marginality respectively.

ENSA II is linked to the state level marginality data by assigning a level of marginality to the state of residence of each individual. ENSA II is not designed to support state level estimation, however. To determine whether each state is represented in ENSA II in proportion to its actual share of the Mexican population, the distribution of ENSA II was compared to the 1990 census and 1995 mid-count data. The ENSA II sample distribution is similar to the census data, except for the PASSPA states, which were over sampled. The final data set for this analysis is comprised of individual level data, but it also includes a contextual level measure, the marginality index at the state level, as well as the regional variables. Table 3 shows which states are included for each region. Dummy variables were created to designate the region where an individual lived. The country was divided in five regions: North, Center, Metropolitan Areas of Mexico City, South and PASSPA. The North region became the reference category.

**Table 3:** Second National Health Survey (ENSA II) regions

**Region**	**States**	**Households (n)**	**Individuals (n)**
North	Baja California, Baja California Sur, Sonora, Chihuahua, Sinaloa, Coahuila, Nuevo León, Tamaulipas, Durango, Zacatecas	2,570	4,905
DF	Mexico City Metropolitan Zone	2,520	5,139
Center	Aguascalientes, Colima, Guanajuato, Jalisco, Michoacán, México, Nayarit, Querétaro, San Luis Potosí, Tlaxcala	2,620	5,225
South	Campeche, Morelos, Puebla, Quintana Roo, Tabasco, Veracruz, Yucatán	2,520	5,227
PASSPA	Chiapas, Guerrero, Hidalgo, Oaxaca	2,520	4,887

PASSPA: Health Services Aid Program for the Uninsured Population

## Results

Table 4 and 5 summarize the sample characteristics and the bivariate relationships to poor self-assessed health and reported morbidity, respectively. Both the urban and the agricultural sectors show class gradients. In the urban sector, 19 percent of the high class reported poor health, while 52 percent of the low class perceived their health as poor. In the agricultural sector, 48 percent of the rural class perceived their health as poor, while only 17 percent of the high rural class reported poor health. This represents almost a three-fold gradient effect for on self-assessed health for both sectors. Regarding reported morbidity, only 5 percent of the individuals who belong to the high urban class reported a health problem, while 15 percent of those belonging to the low urban class reported having a health problem. In the rural sector, 10 percent the low class reported having a health problem, while only 5 percent of the high class declared having a health problem in the past two weeks.

**Table 4:** Characteristics of ENSA II by individuals reporting poor health

Variable	N (%)	N (%) reporting poor health	Chi 2
Male	12304 (48.48)	5242 (32.91)	57.6
Female	13078 (51.52)	5944 (35.87)	
13–15 years of age	2534 (9.98)	656 (25.89)	1.5e^3^
16–30	10568 (41.63)	2980 (28.20)	
31–59	9589 (37.78)	4010 (41.82)	
60–90	2692 (10.61)	1562 (58.02)	
High urban class	631 (4.16)	75 (18.70)	1.1e^3^
Middle high	2500 (16.47)	352 (23.59)	
Middle low	3667 (24.16)	634 (28.51)	
Low urban class	8381 (55.21)	2491 (52.55)	
High rural class	234 (4.86)	6 (16.67)	43.98
Middle class	952 (19.76)	87 (39.01)	
Low rural class	3633 (75.39)	396 (46.10)	
Elementary school incomplete	8258 (33.73)	5943 (39.66)	1.9e^3^
Elementary school complete	5129 (20.95)	2075 (38.35)	
Middle high school incomplete	1961 (8.01)	597 (29.02)	
Middle high school complete	4734 (19.34)	1393 (29.39)	
High school incomplete	965 (3.94)	216 (22.38)	
High school incomplete	1134 (4.63)	270 (23.81)	
College incomplete	2065 (8.43)	408 (19.76)	
College complete	147 (0.60)	17 (11.56)	
Graduate school	91 (0.37)	19 (20.88)	
High marginality	10259 (41.6)	4985 (36.93)	264.6
Medium	9101 (36.9)	4008 (34.52)	
Low marginality	5303 (21.5)	1818 (28.09)	
Bad housing conditions	13242 (52.17)	6997 (39.31)	687.5
Good housing conditions	12141 (47.83)	4189 (28.57)	
North	4905 (19.32)	1643 (26.47)	366.4
Center	5225 (20.58)	2551 (37.61)	
DF	5139 (20.25)	2117 (34.08)	
PASSPA	4887 (19.25)	2406 (36.99)	
South	5227 (20.59)	2469 (36.32)	
0–1 goods	7049 (31.53)	3876 (39.87)	
2–3	5934 (26.54)	2937 (38.42)	898.4
4–5	5357 (23.96)	2154 (32.14)	
6	4018 (17.97)	982 (20.34)	

**Table 5:** Characteristics of ENSA II by individuals reporting any health problem

Variable	N (%)	N (%) reporting any health problem	Chi2 (df)
Male	12304 (48.48)	1198 (7.52)	108.2 (1)
Female	13078 (51.52)	1706 (10.29)	
Age			569.1 (3)
13–15	2534 (9.98)	135 (5.33)	
16–30	10568 (41.63)	657 (6.22)	
31–59	9589 (37.78)	1120 (11.68)	
60–90	2692 (10.61)	564 (20.95)	
High urban class	631 (4.16)	22 (5.49)	159.2 (3)
Middle high	2500 (16.47)	107 (7.17)	
Middle low	3667 (24.16)	154 (6.92)	
Low urban class	8381 (55.21)	734 (15.49)	
High rural class	234 (4.86)	2 (5.56)	21.33 2)
Middle class	952 (19.76)	21 (9.42)	
Low rural class	3633 (75.39)	88 (10.24)	
Elementary school incomplete	8258 (33.73)	1600 (10.68)	332.6 (8)
Elementary school complete	5129 (20.95)	507 (9.37)	
Middle high school incomplete	1961 (8.01)	139 (6.76)	
Middle high school complete	4734 (19.34)	321 (6.77)	
High school incomplete	965 (3.94)	51 (5.28)	
High school incomplete	1134 (4.63)	80 (7.05)	
College incomplete	2065 (8.43)	128 (6.20)	
College complete	147 (0.60)	4 (2.72)	
Graduate school	91 (0.37)	5 (5.49)	
High marginality	10259 (41.6)	1119 (8.88)	3.03 (2)
Medium	9101 (36.9)	1024 (8.82)	
Low marginality	5303 (21.5)	570 (8.81)	
Bad housing conditions	13242 (52.17)	1644 (9.21)	32.38 (1)
Good housing conditions	12141 (47.83)	1260 (8.59)	
North	4905 (19.32)	629 (10.13)	62.4 (4)
Center	5225 (20.58)	668 (9.85)	
DF	5139 (20.25)	437 (7.03)	
PASSPA	4887 (19.25)	582 (8.95)	
South	5227 (20.59)	588 (8.65)	
Goods			
0–1	7049 (31.53)	889 (9.15)	83.92 (3)
2–3	5934 (26.54)	811 (10.61)	
4–5	5357 (23.96)	575 (8.58)	
6	4018 (17.97)	312 (6.46)	

Regional differences were statistically significant. Living in more deprived regions such as PASSPA and the South seem to have a worse perception of their health than less deprived regions. PASSPA is a region specifically defined to target the most underserved geographic areas in terms of public health infrastructure and medical care.

Given that ENSA II has a complex survey design, adjustments were made for clustering and stratification by regions using *STATA 10*. For using survey data, a recent developed test statistic for *STATA *[49] was used to measure goodness-of-fit for these models. Tables 6, 7, 8, 9 show the F-adjusted test statistics. The p-values of all full models suggest no evidence of lack of fit, except for the urban sector. The possession of goods seems to affect the goodness-of-fit. This is probably due to the high correlation between social class and goods possession.

**Table 6:** Urban social class odds ratios (OR) for good self-assessed health

**Variables**	**Model 1**	**Model 2**	**Model 3**	**Model 4**
	**OR** **95 CI**	**OR** **95 CI**	**OR** **95 CI**	**OR** **95 CI**
High class	2.9 (2.1–3.9)	3.4 (2.6–4.5)	3.6 (2.8–4.7)	3.6 (2.8–4.7)
Middle high	2.8 (2.4–3.4)	3.2 (2.7–3.7)	3.3 (2.9–3.9)	3.3 (2.8–3.8)
Middle low	1.8 (1.6–2.1)	1.9 (1.7–2.2)	2.0 (1.8–2.3)	2.0 (1.8–2.3)
Age	0.98 (0.98–0.98)	0.98 (0.97–0.98)	0.98 (0.97–0.98)	0.98 (0.97–0.98)
Gender	1.1 (1.0–1.2)	1.1 (0.9–1.1)	1.1 (1–1.1)	1.1 (1–1.1)
DF	0.6 (0.5–0.8)	0.7 (0.5–0.8)	0.8 (0.7–0.9)	
Center	0.7 (0.6–0.9)	0.8 (0.6–0.9)	0.8 (0.7–0.9)	
North	1.0 (0.8–1.3)	1.0 (0.8–1.3)	1.2 (1.1–1.4)	
High marginality	0.8 (0.6–1)	0.8 (0.6–0.9)		
Medium marginality	0.8 (0.7–0.9)	0.8 (0.7–0.9)		
0–1 goods	0.8 (0.7–0.9)			
2–3 goods	0.8 (0.6–0.9)			

N	9949	11469	11942	11942
LR chi2 (df)	F = 56.8 (12,5932)	F = 76.5 (10,6837)	F = 101.9 (8,7135)	F = 154.99 (5,7138)
LRT	F-adj = 1.35 P = 0.21	F-adj = 2.04 P = 0.03	F-adj = 1.95 P = 0.04	F-adj = 1.56 P = 0.12

Reference categories: Low class, female, low marginality, South, 4–6 goods.

Model 1 = Full model.

Model 2 = Full model minus goods.

Model 3 = Full model minus goods and marginality.

Model 4 = Full model minus goods and marginality and region.

**Table 7:** Agricultural social class odds ratios (OR) for good self-assessed health

**Variables**	**Model 1**	**Model 2**	**Model 3**
	**OR** **95 CI**	**OR** **95 CI**	**OR** **95 CI**
Middle class	1.0 (0.8–1.2)	1.0 (0.8–1.3)	1.2 (0.97–1.4)
High class	1.5 (1.02–2.2)	1.4 (0.9–3)	1.5 (1.02–2.2)
Age	0.98 (0.97–0.98)	0.98 (0.97–0.98)	0.97 (0.97–0.98)
Gender	1.4 (0.9–2.1)	1.4 (0.9–2.0)	1.4 (0.9–1.9)
North	1.5 (1.1–2)	1.6 (1.3–2.0)	
Center	0.9 (0.7–1.2)	0.8 (0.6–1.0)	
PASSPA	0.8 (0.7–1.0)	0.8 (0.7–1.0)	
High marginality	0.7 (0.5–1.1)		
Medium marginality	0.6 (0.4–0.9)		

N	4715	4881	4881
LR chi2 (df)	F = 21.6 (9,3182)	F = 26.9 (7,3305)	F = 37.8 (4,3308)
LRT	F-adj = 1.7 P = 0.08	F-adj = 1.28 P = 0.24	F-adj = 0.81 P = 0.60

Reference categories: High class, Female, South, 0–1 goods, Low marginality.

Model 1 = Full model.

Model 2 = Full model – marginality.

Model 3 = Full model – marginality – region.

**Table 8:** Urban social class odds ratios for reported health problems

**Variables**	**Model 1**	**Model 2**	**Model 3**	**Model 4**
	**OR** **95 CI**	**OR** **95 CI**	**OR** **95 CI**	**OR** **95 CI**
High class	0.4 (0.2–0.5)	0.3 (0.2–0.4)	0.3 (0.2–0.3)	0.3 (0.2–0.3)
Middle high	0.3 (0.3–0.4)	0.3 (0.3–0.4)	0.3 (0.2–0.3)	0.3 (0.3–0.35)
Middle low	0.5 (0.5–0.6)	0.5 (0.4–0.6)	0.5 (0.4–0.5)	0.5 (0.4–0.5)
Age	1.02 (1.0–1.0)	1.02 (1.0–1.03)	1.02 (1.0–1.03)	1.02 (1.02–1.03)
Gender	0.9 (0.8–0.98)	0.9 (0.8–1)	0.9 (0.9–1)	0.9 (0.9–1)
DF	1.5 (1.2–2.0)	1.5 (1.2–1.9)	1.3 (1.1–1.5)	
Center	1.3 (1.0–1.7)	1.3 (1.0–1.6)	1.2 (1.1–1.4)	
North	1.0 (0.8–1.2)	0.9 (0.8–1.2)	0.8 (0.7–0.9)	
High marginality	1.3 (0.98–1.6)	1.3 (1.0–1.7)		
Medium marginality	1.2 (1.0–1.4)	1.2 (1.0–1.4)		
6 goods	1.24 (1.05–1.5)			
2–3 goods	1.33 (1.1–1.5)			

N	9949	11469	11942	11942
LR chi2 (df)	F = 56.8 (12,5932)	F = 76.5 (10,6857)	F = 101.9 (8,7135)	F = 155 (5,7138)
LRT	F-adj = 1.39 P = 0.18	F-adj = 2.05 P = 0.03	F-adj = 1.86 P = 0.052	F-adj = 1.81 P = 0.06

Reference categories: Low class, Female, Low Marginality, South, 0–1 goods.

Model 1 = Full model.

Model 2 = Full model minus goods.

Model 3 = Full model minus marginality minus goods.

Model 4 = Full model minus marginality minus goods minus region

**Table 9:** Agricultural social class odds ratios for reported health problems

**Variables**	**Model 1**	**Model 2**	**Model 3**
	**OR** **95 CI**	**OR** **95 CI**	**OR** **95 CI**
High class	0.7 (0.4–0.97)	0.7 (0.5–1.04)	0.7 (0.4–0.98)
Middle class	1.0 (0.8–1.3)	1.0 (0.8–1.2)	0.8 (0.7–1)
Age	1.02 (1.0–1.02)	1.02 (1.02–1.04)	1.03 (1.02–1.04)
Gender	0.7 (0.5–0.98)	0.7 (0.5–1.01)	0.7 (0.5–1.03)
North	0.7 (0.5–0.9)	0.6 (0.5–0.8)	
Center	1.1 (0.8–1.5)	1.2 (0.98–1.5)	
PASSPA	1.2 (0.96–1.4)	1.2 (0.95–1.4)	
High marginality	1.4 (0.9–2.1)		
Medium marginality	1.6 (1.1–2.2)		

N	4715	4881	4881
LR chi2 (df)	F = 21.6 (9,3189)	F = 26.9 (7,3305)	F = 37.8 (4,3308)
LRT	F-adj = 1.75 P = 0.07	F-adj = 1.36 P = 0.19	F-adj = 0.68 P = 0.72

Reference categories: High class, female, South, low marginality.

Model 1: Full model.

Model 2: Full model – marginality.

Model 3: Full model – marginality and Region.

Table 6 shows a multivariate odds ratio of 2.9 for high urban class individuals perceiving their health as good relative to the low urban class adjusting for age, gender, region, marginality and a proxy for wealth. For the middle high class relative to low urban class, the odds ratio is similar, 2.8, while for the middle low class it was 1.8. All are statistically significant. However, the goods variable seems to be affecting the goodness-of fit due to the high correlation between this variable and social class. Furthermore, some of the other variables specified in the models seem to modify this association. Individuals living in high marginality states are less likely to perceive their health as good as those living in low marginality states.

Table 7 shows the multivariate odds ratios for the effects of agricultural social class on self-assessed health. Except for the OR between high and low rural classes, all were not statistically significant. However, marginality seems to have an effect of its own. Individuals living in PASSPA, the region with the highest marginality index, are less likely to perceive their health as good compared to the South region.

Table 8 does not show a clear gradient effect of belonging to a particular urban social class on reporting a health problem compared to the self-reported health indicator. Only high-class individuals were less likely to report a health problem compared to low class individuals in the fully adjusted model. Including the goods variable, again, does not make a good fit of the full model.

The gradient effects of agricultural social class on reported health problems, shown in Table 9 are not statistically significant. Only being younger or living in the less deprived North region seems to be associated with not having a health problem in the past two weeks.

## Discussion

The social gradient effect on self-assessed health was significant as expected, especially in the urban labour force. This finding is consistent with previous studies that have shown that this health indicator is sensitive to social class differentials [43-45,50]. It is also consistent with the few empirical studies measuring socio-economic gradients in Latin American countries with similar levels of economic development such as Brazil [13-17]. In the only other study conducted in Mexico using social class, the rate of infant mortality decline between 1976 and 1985 was unequal among social classes: 18 percent for the middle-class non-manual workers compared to 12 percent among working class non-salaried workers and only 5 percent decline for the working class salaried workers [17]. Brazil scholars have used socio-economic categories rather than social class indicators. In a recent study, manual workers had a worse perception of their health, compared to non-manual workers [16].

Measuring this gradient effect is important for several reasons. First, reliable, complete, and available data in middle-income countries such as Mexico are not easy to find. These countries do not invest on information systems for routinely collecting data. They mostly rely on surveys to provide strategic information which are conducted depending on resources availability. Second, these middle-income countries, despite being relatively rich compared to several African and Asian countries, suffer from profound social inequalities. Thus, studying these inequalities becomes particularly important to identify the social factors associated with key policy issues such as health and its determinants. Third, the gradient approach explores pathways across the social spectrum, which suggests that social distance among groups such as classes and the lack of social cohesion that this distance creates has an additional negative effect on health beyond material deprivation. It is not only the absence of material resources, but also the lack of political and collective resources available to defend their own interests, which together give rise to a worse perception of health: the lower the social class, the weaker the political position. This means each class faces more constraints towards articulating their demands and defending their own interests as they occupy a lower position [51-54]. However, political participation of the lower social classes may create pressures for governments to respond with supportive public policies such as *Oportunidades*. Evidence exists that *Oportunidades *benefits selected through citizen participation might have been biased in favour of groups with greater potential for collective action. The poorest among the poor are often not capable of organizing to press and demand for public projects, precisely because their deprivation gives them few political, social and civic resources [55].

The gradient pattern in the agricultural labour force for both health outcomes was not as clear as the urban sector. There are several reasons for this. First, most Mexicans that earn their living in agricultural-related economic activities belong to a low social class. ENSA II data show that almost 80 percent belong to this category, while other studies figures range from 89 to 85 percent [56]. Thus, there is less social class variation that reflects in a less evident gradient effect on the two health indicators. This is probably why only health differentials between the high and the low class were statistically significant. Second, the incidence of poverty is higher in rural areas, but inequalities are lower than urban areas. For example, there are 3 poor households without sewage for every non-poor household in rural areas where 25 percent are poor households, while the urban ratio is 5 to 1 where 7 percent are poor [30]. Finally, the rural occupation categories available from ENSA II are not as clearly defined as the urban ones. Thus, social classes are not easily specified for the rural sector. Land owners, for example, are a heterogeneous occupational category in terms of education and wealth.

The results also show evidence that individuals living in the less deprived North region report better health than individuals living in the more deprived South region. This finding is consistent with many studies that have shown that health status is poorer in deprived areas [27].

### Limitations

The cross-sectional character of the data poses some limitations to the interpretation of the results. This type of data is inadequate for fully elucidating the inequality effects on health over the course of a lifetime [57]. However, longitudinal studies have shown that self-assessed health has a considerable predictive validity of mortality, [58] thus providing some insights into how it is that health declines over the life course. Furthermore, there is empirical evidence suggesting that social class differences in morbidity persist through long periods of time. The Whitehall study of British civil servants showed no reduction in the social gradient in morbidity during a twenty years period [11].

The nature of the data available from ENSA II constrained the specification of social class. Although a theoretical framework guided the construction of social class categories, to some extent theory had to be sacrificed in order to obtain empirically testable categories. For example, to better distinguish between middle and high-class categories required information on ownership of the means of production, authority over others in the workplace, and skill exercised in the job. However, none of these characteristics were available. Level of education was then used to sort individuals into the different social classes.

ENSA II is linked to the state level marginality data by assigning a level of marginality to the state of residence of each individual. ENSA II is not designed to support state level estimation, however. This is probably why the results at the state level were not statistically significant. In contrast there were some regions, which are representative of their population, like PASSPA, that showed statistical significance.

Income is another variable that limited the analysis because the non-response rate was very high. A proxy for income, whose validity was tested in another study using ENSAII [47], was employed instead. Although this indicator measures relative deprivation rather than income itself, it was useful to account for its mediating effects in the association between social class and perceived health.

Omitting a relevant variable may bias the estimates of the regression coefficients. The models specified here have not included, for unavailability reasons, some variables that have been identified in empirical studies as important determinants of health. These variables are health behaviour-related variables such as smoking prevalence, alcohol consumption, and nutrition habits as well as physiological variables such as cholesterol levels and obesity, and family history of previous illnesses. Thus, given that these omitted variables have a sound theoretical basis, but are not currently available, the only way to deal with this specification error is to recognize it as a limitation of the analysis.

Multicollinearity is a common problem encountered in identifying the socio-economic factors that influence health. This is because several of the independent variables included in the models such as social class, income, marginality and region are likely to be correlated with each other. All regressions did not show a low tolerance (i.e. values below 0.20), which is an indication that multicollinearity is present [59]. Additionally, analysis of residuals indicated that neither outliers nor influential cases should be of concern.

Finally, future research should perform multilevel analysis as a methodological approach to better measure the effects of the social gradient on health living in different deprivation contexts.

## Conclusion

Overall, the findings of this study provided empirical evidence of how inequality negatively influences health across the social spectrum as well as through area-based policy-related living conditions. Although these findings are not new, they do make an empirical contribution to the scarce Latin American literature in particular, as well as to the social determinants of health literature in general. This is particularly relevant for Mexico and other countries that do not publish empirical findings because they lack good, reliable, and complete data. This suggests a call for governments to invest in reliable, complete and integral information systems for better decision making and enhanced empirical research.

Measuring the effects of social inequality on health poses important challenges in Mexico and similar middle-income countries. Social and political variables are rarely included in health surveys, much less in routinely gathered information. The two latest National Health Surveys conducted in Mexico in 2000 and 2006 have focused on the biological and behavioural aspects of health, neglecting key variables to measure social class such as occupation and social position.

The social gradient effect on health has important policy implications because it suggests that public policies should address inequalities by tackling the systematic differences in life chances, living standards and lifestyles associated with people's unequal positions in the socio-economic hierarchy, not only in the deprived living conditions of the lower social classes [60]. The social gradient effect suggests that more inclusive programs would be more beneficial to the lower classes and the whole social spectrum in general rather than programs targeted to the most deprived.

Targeting the poor may therefore not be the best policy solution to overcome structural inequality as opposed to more inclusive redistribution policies, especially when accurate targeting is so difficult to achieve and may exclude many poor people. An evaluation of the targeting performance of *Oportunidades*, which currently covers approximately 5 million poor households based on proxy-means testing in Mexico, indicates that there is substantial under coverage of poor households, with only 45 percent of eligible poor households receiving its benefits [61]. However, *Oportunidades *has been successful in reducing poverty defined as the population that is deprived from acceptable minimum food consumption. *Oportunidades *has yet to overcome the poverty which deprives Mexicans from being well-educated and healthy.

The negative effects on health of living in a highly deprived region such as PASSPA were consistent with many studies that have shown that health is worse in deprived areas [27-29,62-64]. Recent studies as well as the WHO Commission on Social Determinants of Health suggest that policy-oriented indicators should be included in studies assessing the effects of social inequalities on health [63,64]. Furthermore, although the marginality index was specifically designed for the Mexican context, it suggests that similar indicators may be used to measure area-based policy related living conditions.

## Competing interests

The author declares that they have no competing interests.
